# Expérience sénégalaise de la radiothérapie par arc-thérapie volumétrique dans le traitement des adénocarcinomes rectaux localement avancés: étude rétrospective de 59 cas

**DOI:** 10.11604/pamj.2026.53.107.47465

**Published:** 2026-03-02

**Authors:** Kanta Ka, Mamadou Moustapha Dieng, Lionel Eric Francis Edimo, Boucar Ndong, Adji Thioro Boye, Gora Thiaw, Bénédicte Souho, Alpha Oumar Touré, Papa Macoumba Gaye, Abdou Aziz Kassé

**Affiliations:** 1 Service de Radiothérapie, Centre Hospitalier National Dalal Jamm, Guédiawaye, Sénégal,; 2 Centre International de Cancérologie de Dakar, Dakar, Sénégal,; 3 Service de Radiothérapie, Hôpital Général de Douala, Douala, Cameroun,; 4 Service de Médecine Nucléaire, Centre Hospitalier National Dalal Jamm, Guédiawaye, Sénégal,; 5 Service de Chirurgie Générale, Centre Hospitalier National Dalal Jamm, Guédiawaye, Sénégal

**Keywords:** Cancer du rectum, VMAT, radiothérapie, radiochimiothérapie, Afrique, Sénégal

## Aux éditeurs du Pan African Medical Journal

Le cancer colorectal connaît une progression constante à l'échelle mondiale, avec près de 1,93 million de nouveaux cas estimés en 2022 [1]. En Afrique subsaharienne, son incidence demeure inférieure à celle rapportée dans les pays à hauts revenus, mais il représente un enjeu croissant en raison du diagnostic souvent tardif et des contraintes d'accès aux soins spécialisés [1]. Au Sénégal, les cancers colorectaux figurent parmi les cancers fréquents, avec une mortalité non négligeable [1].

La prise en charge du cancer du rectum repose aujourd'hui sur une approche multimodale associant radiothérapie préopératoire, chimiothérapie et chirurgie, afin de réduire la récidive locale, d'optimiser le downstaging tumoral et d'augmenter la conservation sphinctérienne [2-4]. L'arc-thérapie volumétrique modulée (VMAT) permet une meilleure conformation de dose et une meilleure épargne des organes à risque par rapport à la radiothérapie conformationnelle tridimensionnelle (RC3D), pouvant se traduire par une toxicité moindre, tout en maintenant l'efficacité oncologique [5]. Toutefois, les données issues de contextes africains demeurent limitées. Nous rapportons l'expérience sénégalaise au Centre international de cancérologie de Dakar (CICD) chez des patients traités par radiochimiothérapie préopératoire utilisant la VMAT.

L'objectif était d'évaluer, en conditions réelles, les caractéristiques des patients, les résultats précoces (réponse clinique, réponse pathologique, chirurgie, conservation sphinctérienne) et la tolérance aiguë de la VMAT chez des patients atteints d'adénocarcinome rectal localement avancé (Tableau 1).

**Tableau 1:** caractéristiques des patients, résultats précoces et tolérance aiguë de l’arc-thérapie volumétrique modulée

Variables		N (%)
Sexe		
Masculin		29 (49,2)
Féminin		30 (50,8)
Age, médiane (extrêmes)		56 (23 -92)
Localisation		
Bas rectum		24 (40,7)
Moyen rectum		28 (47,5)
Haut rectum		7 (11,9)
Classification		
T1-T2		16 (27,2)
T3-T4		43 (72,8)
Traitements		
Chirurgie		53 (72,9)
Chimiothérapie concomitante		52 (88,1)
Radiothérapie		
Dose	45 Gy	9 (15,3)
	50 Gy	39 (66,1)
	50,4 Gy	11 (18,6)

Nous avons mené une étude rétrospective descriptive et analytique monocentrique au CICD, incluant tous les patients traités entre le 1er janvier 2020 et le 31 décembre 2023. Étaient inclus les patients ≥ 18 ans ayant un diagnostic histologique d'adénocarcinome rectal localement avancé (cT3-T4 et/ou N+), sans métastase à distance au diagnostic, et traités par radiochimiothérapie préopératoire utilisant la VMAT. Étaient exclus les patients métastatiques, ceux traités à visée palliative, ceux irradiés par une autre technique (2D/RC3D), et les dossiers incomplets. La radiothérapie était délivrée sur un accélérateur linéaire (Elekta®) à la dose totale de 45 à 50,4 Gy en 25 à 28 fractions (1,8-2 Gy/fraction). Une chimiothérapie concomitante (capécitabine ou 5-FU) était administrée selon l'état clinique et les disponibilités. Les toxicités aiguës étaient codées selon CTCAE v5.0.

Au total, 59 patients ont été inclus. L'âge médian était de 56 ans (23-92) et la répartition par sexe était quasi équilibrée (49,2% d'hommes). Le niveau socio-économique bas était retrouvé chez 81,4%. La rectorragie constituait le principal symptôme (81,4%). Les tumeurs siégeaient majoritairement au moyen rectum (47,5%) et au bas rectum (40,7%). Le stade le plus fréquent était T3N1. Une IRM pelvienne de stadification a été réalisée chez 86,4% des patients. Les doses les plus utilisées étaient 50 Gy (66,1%) et 50,4 Gy (18,6%), alors que 45 Gy a été délivrée chez 15,3%. Une chimiothérapie concomitante a été administrée chez 88,1%.

La chirurgie a été réalisée chez 72,9% (n=53). Le taux de conservation sphinctérienne était de 53,4%. Après radiochimiothérapie, une régression tumorale clinique (complète ou partielle) a été observée chez 71% des patients. Chez les patients opérés, une réponse pathologique complète (pCR) a été observée dans 16,3% des cas, et des marges R0 ont été obtenues chez 86%. La tolérance au traitement a été globalement favorable: les toxicités aiguës rapportées étaient principalement digestives (49%), urinaires (37%) et cutanées (32%), toutes de grade 1-2, sans toxicité ≥ grade 3. Ces résultats sont cohérents avec l'intérêt dosimétrique de la VMAT [5].

Notre étude présente des limites liées à sa nature rétrospective, à la taille de l'échantillon et à l'absence de suivi prolongé permettant d'analyser la récidive locale et la survie. Néanmoins, elle documente une mise en œuvre effective d'une technique moderne de radiothérapie (Tableau 2), dans un contexte africain, avec des résultats précoces de l'ordre de grandeur de la littérature internationale [2-4], et une excellente tolérance aiguë.

**Tableau 2:** résultats oncologiques et toxicité de la radiothérapie

Variables	N (%)
Réponse clinique post- RCC	
complète	10 (16,9 %)
partielle	32 (54,2 %)
nulle	17 (28,8)
Qualité de la chirurgie	
R0	46 (86)
R1	7 (14)
Réponse pathologique post-RCC	
complète	9 (16,3)
partielle	35 (59,3 %)
nulle	15 (24,4 %)
Toxicités de la radiothérapie par VMAT	
Digestives	29 (49)
Urinaires	22 (37)
Radiodermite	19 (32)

Abréviations: VMAT = arc-thérapie volumétrique modulée; RCC = radiochimiothérapie; pCR = réponse pathologique complète

### Conclusion

La radiochimiothérapie préopératoire utilisant la VMAT dans les adénocarcinomes rectaux localement avancés apparaît réalisable au Sénégal, avec une excellente tolérance aiguë et des résultats précoces encourageants, notamment en termes de régression tumorale (71%), de résection R0 (86%) et de conservation sphinctérienne (53,4%). Cette étude met en évidence, en conditions réelles et en contexte à ressources limitées, la faisabilité de l'implémentation d'une technique avancée de radiothérapie avec un profil de tolérance favorable et des résultats cohérents avec les données de la littérature. Elle souligne également l'importance du développement de plateaux techniques modernes et d'une prise en charge multidisciplinaire structurée dans l'amélioration des résultats oncologiques en Afrique subsaharienne.

## Remerciements

Nous remercions les équipes du CICD et du CHN Dalal Jamm impliquées dans la prise en charge des patients et la gestion des dossiers médicaux.
